# The Effectiveness and Validity of Inspiratory Muscle Training in the Training Process of Disabled Swimmers

**DOI:** 10.3390/jcm13185365

**Published:** 2024-09-10

**Authors:** Paulina Okrzymowska, Wojciech Seidel, Krystyna Rozek-Piechura

**Affiliations:** 1 Department of Physiotherapy in Interal Medicine, University of Health and Sport Sciences, 51-612 Wroclaw, Poland; krystyna.rozek-piechura@awf.wroc.pl; 2 Department of Paralympic Sport, University of Health and Sport Sciences, 51-612 Wroclaw, Poland; wojciech.seidel@awf.wroc.pl

**Keywords:** respiratory muscle training, respiratory muscles, exercise physiology, clinical practice, disabled athletes

## Abstract

**Objectives:** The aim of this study was to evaluate the effect of medium-intensity inspiratory muscle training added to standard swimming training on inspiratory muscle strength and aerobic endurance levels and training status in disabled swimming athletes. **Methods:** This study involved 16 disabled athletes: group I—athletes performing swimming training with 8 weeks inspiratory muscle training—IMT (50% of the maximum inspiratory pressure); group II—athletes performing standard swimming training with 8 weeks inspiratory muscle training (15% MIP). The following tests were performed three times: MIP, MEP, Borg RPE scale and swimming test: T-30 test; 8 × 100 progressive test. **Results:** There was a significant increase in the MIP and MEP in group I after IMT. There was a significant increase in the distance swam during the T-30 only in group I after IMT. Significant differences were found between the first and third measurements in group I, and the effect was maintained at follow-up. There was also a significant reduction in La concentration in group I after IMT. There was a significant reduction in heart rate at 88% and 93% of the maximum speed in group I after the applied training. **Conclusion:** Inspiratory muscle training with 50% of the maximum inspiratory pressure load significantly increased the respiratory muscle strength of disabled swimmers. The application of higher-intensity IMT effectively improved the training level and physiological parameters of the swimmers’ effort, indicating the need to include this type of training in the standard preparation of disabled swimmers.

## 1. Introduction

Respiratory muscle fatigue impairs exercise capacity through an increased perception of shortness of breath [1,2], and inspiratory muscle fatigue before full-body exercise results in a 15% reduction in exercise tolerance, regardless of sex [3]. Reducing the supply of oxygen to the active muscles of the lower limbs may lead to accelerated quadriceps fatigue and the premature termination of a given physical activity. The available literature indicates that during long-term, high-intensity physical exercise, the efficiency of peripheral muscles decreases due to the increased work of respiratory muscles and shortness of breath. This, in turn, may contribute to respiratory muscle fatigue and reduced global performance [4].

During swimming, the breathing pattern is altered in relation to the breathing pattern on land. The breathing pattern during swimming is synchronised to the swimming rhythm and results in a forced inspiratory phase. Forced breathing phases can have a significant impact on breathing frequency (fb) and tidal volume (VT). In the long term, this can lead to varying degrees of hypoxaemia and hypercapnia. Compared to exercise on land, the higher tidal volume seen during swimming is probably related to the varying respiratory rhythm, the hydrostatic pressure exerted on the chest. The change in ventilation mechanics is altered in swimmers due to upper and lower limb movement for propelling and intermittent facial immersion [5]. Such a change has also been observed among disabled swimmers. In a study by Cavaggioni et al., a significant difference was found in breathing pattern, trunk stability and upper body power variables at the peak of the training season [6].

It is noteworthy that swimming is one of the sports that causes the most respiratory muscle fatigue, highlighting the need for athletes to have good respiratory muscle strength and endurance [7]. The relevance of respiratory training has not been fully assessed among Paralympic swimmers [8], and there is a reason to carry out an evaluation of the effectiveness of this type of training. Traditional sports training does not provide sufficient stimulus to improve respiratory muscle function, and there has been a rationale for the introduction of respiratory muscle training [8].

Additionally, reduced respiratory function may reduce performance during high-intensity exercise due to the activation of the metaboreflex mechanism. This mechanism, in response to inspiratory muscle fatigue and dyspnoea, results in an increase in blood flow through the respiratory muscles and a decrease in blood flow to the active peripheral muscles [9]. The available literature indicates that diaphragmatic fatigue (DF) induced by the sympathetically mediated metaboreflex may result in increased heart rate, blood pressure and limb vascular resistance. Reducing the occurrence of the metaboreflex of the inspiratory muscles may improve the haemodynamics of the limb and respiratory muscles, which may ultimately improve the efficiency of sports training [1]. Therefore, the use of inspiratory muscle training (IMT) as a potential method to minimise the activation of this mechanism is justified [10]. At the same time, the greater resistance of the inspiratory muscles contributes to maintaining a constant blood supply and perfusion within them, which directly reduces their metabolic demand, leading to better performance and functionality [11]. Hellyer et al. confirmed that diaphragm fatigue reduces oxygen delivery to the muscles of the musculoskeletal system, which affect exercise performance [12]. Segizbaeva et al. assessed the effects of inspiratory muscle training (IMT) on the fatigue resistance of the diaphragm (D), sternocleidomastoid (SCM) and scalene (SC) muscles. The maximum inspiratory pressure and EMG analysis were used as indicators to assess inspiratory muscle fatigue in all subjects before the IMT intervention; a reduction in the MIP level and centre of gravity (fc) frequency in the EMG power spectrum (the D, PS, SCM and SC muscles) was observed pre–post exercise (*p* < 0.05). Such changes were not observed after IMT. The study showed that in healthy people, IMT causes a significant increase in the MIP (+18%), a delay in inspiratory muscle fatigue during strenuous exercise and a significant improvement in maximum work efficiency. According to the authors, IMT increases resistance to the development of inspiratory muscle fatigue during high-intensity exercise [13].

Among several well-known training methods aimed at improving physical performance, such as training periodisation and technical and tactical training, inspiratory muscle training (IMT) has been used in various Olympic and Paralympic disciplines [14]. Although the effect of IMT on increased performance is still debated, some studies have shown positive benefits in the way a wheelchair is used [14].

Swimming is one of the most popular sports in the Paralympics [15]. Swimming is useful and very popular among people with disabilities [16], so the topic addressed is extremely important for the wider population.

The available literature contains few works on the use of inspiratory muscle training in disabled people practising professional sports. These works mainly concern basketball players with spinal cord damage who use wheelchairs [17,18]. Additional knowledge on the effectiveness and appropriateness of inspiratory muscle training in disabled athletes is lacking. The current state of knowledge is still limited in the area of assessing the persistence of effects after completing inspiratory muscle training. The available literature also lacks a confirmation of the validity of inspiratory muscle training as a method complementing sports training programs, leading to the best possible sports results for athletes with disabilities.

There are reports in the literature of an increasing prevalence of abnormalities in normal respiratory function associated with changes in their musculoskeletal system [19,20]. It is therefore reasonable to include additional training to improve respiratory function and strengthen the respiratory muscles of disabled athletes [21].

At the same time, it is worth noting that swimming for both non-disabled and disabled swimmers should be aimed at developing muscle strength to improve overall performance. In disabled swimmers, the level and nature of musculoskeletal impairment may affect the efficiency of muscle force generation and thus swimming speed, showing more asymmetry [22,23]. The type and level of physical impairment (i.e., high and low range) may affect force production and swimming speed, showing more asymmetry [24]. The available literature indicates that inspiratory muscle training in disabled athletes helps to improve thoracic ranges of motion by improving respiratory muscle strength. Indirectly, IMT may improve trunk muscle strength [25].

Therefore, the aim of this study was to assess the impact of medium-intensity inspiratory muscle training added to standard swimming training on the strength of respiratory muscles and the level of aerobic capacity as well as the training status of disabled swimmers.

## 2. Materials and Methods

This research covered 16 players (men) of the Polish Disabled Sports Association “Start”. The eligibility of the players for this research program was determined by the team coach and the head of the research experiment based on specific inclusion criteria. This study was conducted according to the guidelines of the Declaration of Helsinki, and approval was obtained from the Ethics Committee of the Wroclaw University of Health and Sport Sciences on 15 March 2019 (no. 17/2019). The ethics committee reviewed the entire experimental protocol before approval. All experimental protocols, research methods, statements to subjects and consents to participate in this project were approved by the designated ethics committee.

To form the most homogeneous group of respondents possible, criteria were established for including and excluding players from this study. The inclusion criteria included belonging to the national team of the Polish Disabled Sports Association “Start” and being diagnosed with musculoskeletal dysfunction (swimming class: S6-10, SB6-10, SM6-10). Swimming classes were granted by the classifier International Paralympic Committee. Each respondent provided informed consent to participate in this research (in the case of minor participants, additional informed consent was obtained from parents/legal guardians).

The exclusion criteria included coexisting respiratory diseases, mental disorders that could prevent contact and cooperation and conditions that developed after recent damage to the eardrum.

The subjects were divided into 2 groups, and the allocation to groups was randomised. Probability distribution tables using random number generations were used to randomise the subjects:

− Group I (IMT group)→Competitors training according to the standard swimming training model and additionally supplemented with medium-intensity inspiratory muscle training at a load level of 50% of the MIP.
− Group II (sham-IMT group)→Competitors training according to the standard swimming training model and additionally supplemented with low-intensity inspiratory muscle training at a load level of 15% of the MIP, in accordance with the literature indicating the need to use a minimum load in the control group, meeting the time requirements of the training used in both groups [17].

All subjects underwent eight weeks of inspiratory muscle training using personal PowerBreath KH1 devices. The training took place at home four times per week and once under the supervision of a trainer. Swimming training was conducted under the supervision of the team coach, taking the degree and type of dysfunction and the player’s sports level into account. Detailed characteristics of the subjects in both groups are presented in Table 1, along with the significant differences between the groups (t-*t* test and chi-square test).

**Figure 1:**
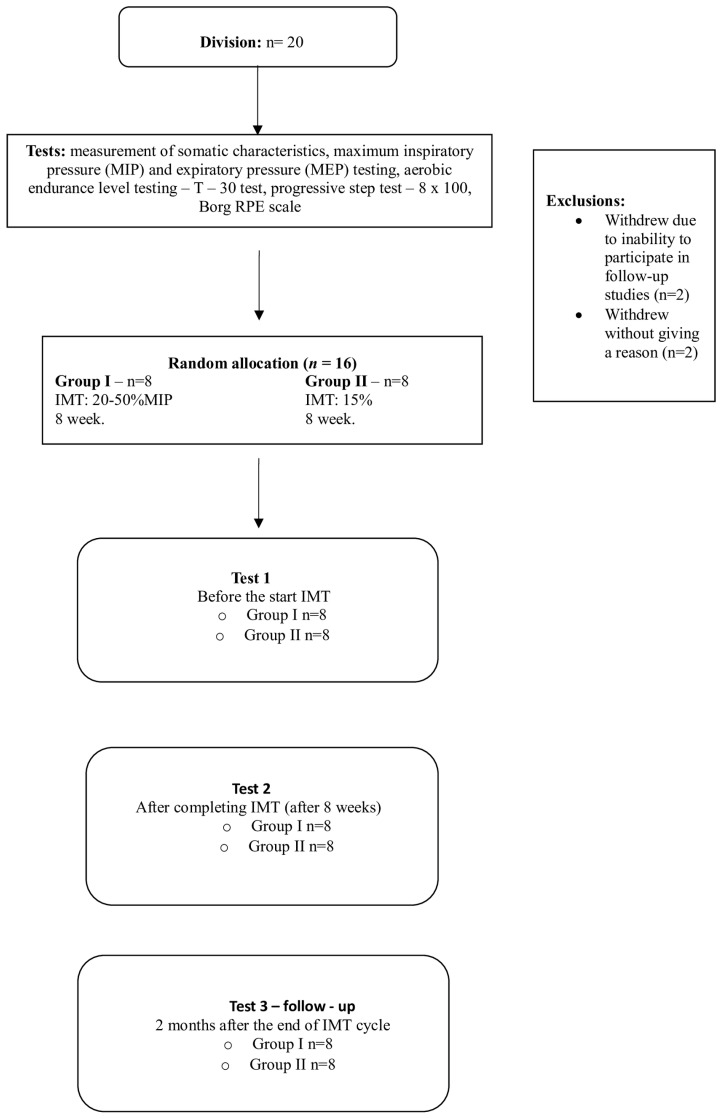
The design of the experiment (present work).

All tests were carried out at 3 time points: I—before the summary of the inspiratory muscle training; II—after the analysis of the inspiratory muscle training (8 times) and III—after the main swimming exercises were performed (approximately 2 activities after the routine cycle of inspiratory muscle exercises: follow-up) (Figure 1).

The respiratory muscle strength test was performed using Jaeger’s MasterScreen Pneumo spirometer (CareFusion, Wurmlingen, Germany) and a special pneumatic attachment, in accordance with ATS/ERS guidelines. The standard procedure requires taking 5 to 10 correct measurements. The acceptable difference is less than 5% or 5 cm H_2_O [26]. The absolute values measured during this study referred to normalised values [27], which are the values of individual lung function variables calculated hypothetically based on morphometric parameters such as sex, age, height and, to a lesser extent, body weight. The regression formula inputted into the Jaeger Electronic Masterscreen spirometer to calculate the normal values corresponded to the standards developed by the European Community for Coal and Steel, Luxemburg [28].

### 2.1. Swimming Test

The examination of the players’ training levels (T-30 test) was conducted by a coach under strict supervision. Before starting the test, the competitors performed a 15 min warm-up on the edge of the pool (50-metre pool) and an approximately 20 min warm-up in the water. Both warm-ups were carried out according to the coach’s instructions. The T-30 test involved freestyle swimming for the longest possible distance in 30 min [29,30].

The total distance swam (DST) was taken into account for the analysis. The coach determined the swimmers’ individual positions using a measuring tape to the nearest 1 metre from the wall of one fixed end of the pool [29].

To assess the players’ training status, aerobic endurance and the effects of the inspiratory muscle training, a progressive 8 × 100 freestyle or first style test was used in a 50-metre pool.

The selection of swimming speeds and the percentage distribution of the intensity of the given 100-metre sections were determined at five designated implementation levels. The selection was made individually for each player as follows [31]:

− Level I—swimming 3 times the distance of 100 m in the starting time, i.e., approximately 77% in relation to the best result obtained in the competition in a given training period. Exercise at this level can be treated as a warm-up for the stimulation of individual functional mechanisms of the body for further work.
− Level II—swimming 2 sections of 100 m with an intensity of approximately 83% in relation to the best result.
− Level III—swimming 100 m at a speed of 88% of the maximum speed.
− Level IV—swimming 100 m at a speed of 93% of the maximum speed.
− Level V—swimming 100 m at the maximum intensity.

During the test, the heart rate (HR [bpm]) and blood lactate formation concentration [mmol/L] were measured. HR measurement was performed at rest and after swimming subsequent 100 m sections (i.e., three measurements at level 1, two measurements at level 2 and one measurement at levels 3–5). POLAR sports testers were used to assess heart rate.

The lactic formation (La) concentration was assessed using the Lactate Scout device (Lactate Scount+, Leipzig, Germany) before starting the test and during rest breaks between individual levels in the following order: immediately after swimming the third 100 m section, 1 min after swimming the fifth 100 m section, 3 min after swimming the sixth 100 m section and 1 and 3 min after swimming the seventh section (at 3, 6 and 9 min for women; at 4, 7 and 10 min for men) after swimming the last 100 m section. The duration of rest breaks between individual sections at subsequent levels was 1 min, that between the first and second and second and third levels was 3 min, that between the third and fourth levels was 5 min and that between the fourth and fifth levels was 20 min [30,31].

The 8 × 100 protocol is considered suitable for assessing the training effects of swimming, as it stimulates effort levels close to the maximum VO2, as well as high blood lactate concentrations [32].

### 2.2. Intervention

Inspiratory muscle training (IMT) was added to the players’ daily training cycles and was conducted for eight weeks using an inspiratory muscle trainer (PowerBreathe KH1-Series; POWERbreathe International Ltd., Southam, UK). Due to the scale of the PowerBreathe device, the obtained MIP measurements (kPa) were converted to the device unit (H_2_O) as follows: 1 kPa = 10.2 cm H_2_O.

Inspiratory muscle training was conducted 4 times per week at home and once under the supervision of a trainer. Competitors in group I performed IMT twice per day (morning and evening). The players in group II trained once per day in the morning. To control the training, each participant needed to complete a training diary [33].

Group I performed 30 dynamic breaths at the threshold values of the MIP. The established loads determined the safety of the training. In addition, training sessions took place twice per day (in the morning and after afternoon training) (Table 2). Competitors in group II performed 60 breaths at a training at a constant load of 15% of the MIP [33,34] (Table 3).

### 2.3. Sports Training Program

Swimming training covered the period of the early competitive season, i.e., the general preparation phase. All swimming training sessions took place in 50 m pools. Each athlete had standardised swim training. The training load distribution used was an average training volume: approximately 5000–6000 m: approximately 20% in the REC zone, 50% in the basal endurance zone (EN1) and threshold endurance zone (EN2), 20% in the overload endurance zone (EN3) and approximately 10% at the anaerobic level—lactate production (SP1) and sprint level (SP2). In addition, the players also trained on land with a training program aimed mainly at strengthening all groups (strength exercises using resistance bands, their own load or a partner’s load and functional, flexibility and coordination exercises).

### 2.4. Statistical Analyses

The results of this study were statistically analysed using the STATISTICA PL V.12.0 programme. The arithmetic mean and standard deviation were calculated in basic descriptive characteristics for measurable traits. To assess the homogeneity of the studied groups, the t-test was applied for quantitative characteristics and the chi-square test of concordance for qualitative characteristics. After checking the normality of the distribution with the Shapiro–Wilk test, it was decided to use an ANOVA with repeated measures and a post hoc (LSD—Least Significant Difference) test. Multiple regression analysis was performed to determine the effect of explanatory variables on the obtained swimming performance score measured by the T-30 test (explained variable). The values of tests and coefficients at the *p* < 0.05 level were assumed to be statistically significant and are highlighted in this paper in bold.

## 3. Results

The statistical analysis began with the assessment of changes in the maximum inspiratory pressure (MIP) parameter.

Based on the analysis of variance (Table 4), a significant increase in the MIP value was found, both in kPa and in the percentage (%) of the predicted value in group I after IMT. Moreover, statistical analysis showed a significantly higher MIP value in kPa and the percentage predicted value in Study 2 in the study group than in the sham group. Statistical analysis showed statistically significant differences between the second and third measurements in the study group.

A significant increase in the MEP value, both measured in kPa and in the percentage of the predicted value, was found in the study group after IMT (Table 5). At the same time, a significantly higher MEP percentage predicted value in Study 2 was found in the study group than in the sham group. Statistical analysis showed significant differences between the first and third measurements in the study group, and the effect was maintained in the follow-up study.

The assessment of changes in the distance swam during the fitness level test (the T-30 test) based on the analysis of variance (Table 6 and Table 7) showed a significant increase in the distance swam only in the study group after IMT. Moreover, statistically significant differences were demonstrated between the first and third measurements in the study group, and the follow-up effect was maintained.

A significant decrease in the lactic formation concentration (mmol) was observed after swimming 100 m at swimming speeds of 77%, 83%, 88% and 93% of the maximum speed and after swimming at the maximum speed in the study group after IMT (Table 6 and Table 7). However, in the sham group, after 8 weeks, a significant increase in the blood lactate formation concentration (mmol) was observed after swimming 100 m at swimming speeds of 77%, 83% and 88% of the maximum speed. Moreover, significant differences were found between the first measurement and the third measurement of the blood lactate formation concentration after swimming 100 m at a speed of 83% of the maximum speed in the sham group, and the follow-up effect was maintained.

There was also a significant reduction in heart rate after swimming 100 m at a speed of 77% of the maximum speed only in the sham group after the training period (Table 6 and Table 7).

A significant reduction in heart rate was observed after swimming 100 m at a speed of 88% of the maximum speed in the study group after training. In the sham group, at a given speed, a significant increase in the assessed parameter was observed after 8 weeks. Moreover, significant differences were demonstrated between the first measurement in the sham and study groups and the third measurement in the sham and study groups, and the follow-up effect was maintained.

At the same time, a significant decrease in heart rate was found after swimming 100 m at a speed of 93% of the maximum speed in the study group after training. In the sham group, a significant increase in this parameter was observed after 8 weeks. Moreover, statistical analysis showed significant differences between the first measurement in the study and sham groups and the third measurement in the study and sham groups, and the follow-up effect was maintained.

## 4. Discussion

Inspiratory muscle training at an intensity of 50% MIP significantly improved the training level of the swimmers tested, which was associated with an increase in swimming distance in the T-30 test performed. The introduction of inspiratory muscle training (50% MIP) significantly reduced blood lactate formation levels and significantly lowered heart rate (at 88% and 93% of maximum speed) in the 8 × 100 progressive test. The application of medium-intensity inspiratory muscle training (50% MIP load) effectively improved the training level and physiological parameters of the swimmers’ effort.

An inspiratory muscle training protocol with progressive loading was found to be more effective in increasing aerobic capacity in a group of disabled basketball players [19]. The same observation can be seen in the present study. After 8 weeks of IMT training in the group with a progressive load of 20% to 50% of the MIP, there was a significant improvement in the training level and favourable changes in the physiological exercise parameters of the assessed swimmers. Such a significant change was not observed in the IMT group performing training at a constant 15% MIP. As reported in the literature, IMT training loads from moderate to high MIP resistance (50–80% MIP) can reflect significant changes in respiratory parameters [35,36,37]. It is also important to note that in patients and people with disabilities, when using IMT at a medium load level, we can achieve more work of breathing and a higher inspiratory volume with less strenuous muscle involvement [38]. A load of 15% MIP resistance at 60 breaths once a day is considered a placebo group. It is also important to note that, according to the literature, a load of 15% MIP causes little change in respiratory muscle function among both healthy subjects and subjects with disabilities [14,19,34].

In sports disciplines, special devices for training the respiratory muscles, and in particular, the inspiratory muscles, seem to be useful to improve results, mainly due to the reduction in the metaboreflex effect, the feeling of fatigue and shortness of breath. The use of this type of training may be recommended in various sports due to the high dose–response relation. According to Lorca-Santiago et al. (2020), the use of IMT in daily sports practice four to seven times per week is an effective protocol [39]. The results of our own research also showed a highly significant increase in the MIP value of disabled swimmers after 8 weeks of inspiratory muscle training. It was important that the obtained training effect was maintained 2 months after the end of IMT in the study group, as opposed to the sham group, in which minimal load training was used.

Inspiratory muscle training (IMT) is a complementary method that can improve sports performance. Previous research has shown that IMT reduces the feeling of shortness of breath, lactate accumulation and peripheral fatigue and thus improves the effectiveness of exercise [14,40,41]. Inspiratory muscle training (IMT) is important in reducing the perception of respiratory and peripheral fatigue, which may consequently improve neuromuscular performance. Such changes may result in greater improvements in exercise capacity than sports training alone. This was confirmed by our own research, which showed a significant reduction in the perception of the severity of exercise measured by the Borg scale.

Cavalcante Silva et al. assessed the effect of IMT on exercise tolerance, repeated sprint ability (RSA), maximum inspiratory pressure (MIP) and peak inspiratory flow (PIF) in football players. The study included 22 healthy football players from the first league of football. A 50% MIP load was used with a 2-week period of IMT. The authors found a highly significant reduction in sprint time after IMT. In addition, the researchers observed a highly significant reduction in the following parameters after IMT: the total sprint time and percentage (RSA % dec). Additionally, they observed higher peak inspiratory pressure and peak inspiratory flow results after just a 2-week IMT period, which was highly significant. According to the authors, the increase in the efficiency of the inspiratory muscles led to a reduction in sprint time and improved exercise tolerance [41]. A similar tendency was confirmed by our own research, in which in the group performing IMT with a load of 50% of the MIP, a significant extension of the distance swam was achieved during the performance level test (T-30 test) as well as a reduction in the perception of the intensity of the exercise.

Guy et al. assessed the impact of IMT on exercise tolerance among football players. Thirty-one men were randomly assigned to one of three groups: the experimental (EXP: n = 12), placebo (PLA: n = 9) and control (CON: n = 10) groups. The EXP and PLA groups completed a 6-week sports training program with the simultaneous use of IMT. The control group did not perform IMT or soccer training. All participants underwent tests before and after 6 weeks of training, including the following assessments: spirometry tests, maximum inspiratory pressure measurements, a multistage MSFT fitness test and a football-specific SSFT fitness test. The experimental group achieved significant improvement in the MIP after 6 weeks of training. At the same time, the study did not show any significant changes in the FVC and FEV1 parameters in any of the studied groups. The fact that this group showed a significant increase in the distance covered in a multistage fitness test is important. Interestingly, the experimental group achieved significantly lower blood lactate concentrations after the fitness test. Adding IMT to preseason football training improved exercise tolerance (MSFT distance covered) and reduced the lactate concentration [42]. In our study, improvement was achieved in most of the assessed functional parameters of the respiratory system in the study group. It is also important to note that this group also achieved a significant increase in the distance covered during the T-30 test. At the same time, a significant reduction in blood lactate formation at all stages of the 8 × 100 progressive test was observed.

Ohya et al. attempted to explain the effect of 6 weeks of high-intensity inspiratory muscle strength training (IMST) on the MIP and swimming performance in highly trained competitive swimmers. The study included thirty male competitive swimmers who were assigned to high-intensity IMST (HI; n = 10), medium-intensity IMST (MOD; n = 10) and control groups (n = 10). The training intervention consisted of twice-daily sessions for 6 days per week at inspiratory pressure threshold loads equivalent to 75% of the MIP (HI) and 50% of the MIP (MOD). The authors assessed the MIP and swimming performance before and after the intervention. Swimming performance was assessed in timed swimming tests and 100 m freestyle tests in a 25 m long pool with free and controlled breathing. For rate-controlled breathing, participants took one breath every six beats. The MIP values in the HI and MOD groups were significantly higher after 2 and 6 weeks of IMST than before IMST. Frequency-controlled 100 m freestyle swim times were significantly shorter after IMST than before IMST in both the HI and MOD groups. According to the researchers, an inspiratory pressure threshold load corresponding to 50% of the MIP may be sufficient to significantly improve the MIP and swimming performance in highly trained competitive swimmers [43]. The same change was observed in our own research using the same threshold load. This experiment showed a significant increase in the distance swam and the strength of the respiratory muscles (MIP, MEP).

Brown et al. examined the effects of inspiratory muscle training on the volitional increase in blood lactate levels ([lac(-)](B)) during cycling and the kinetics of blood lactate and oxygen uptake at the beginning of training. They studied twenty men who were divided into two groups: the IMT group and the control group. Before and after the 6-week intervention, they administered two repeated 30 min constant-power MLSS tests. The first trial was a reference trial, and during the second trial, which lasted for 20 to 28 min, participants imitated a breathing pattern proportional to 90% of the maximum minute ventilation of the increasing exercise test. The IMT group performed 30 consecutive dynamic breaths twice per day for 6 weeks using the POWERbreathe trainer. They adjusted the training load individually, and it amounted to 50% of the MIP. After the intervention, the maximum inspiratory pressure significantly increased by 19% only in the IMT group (*p* < 0.01). Moreover, only in this group did the time constants of blood lactate kinetics and phase II oxygen uptake at the beginning of exercise and during MLSS significantly decrease. According to the authors, the changes obtained were the result of an increase in the ability of inspiratory muscles to transport oxygen and/or lactate under the influence of IMT [44]. In our own study, in the experimental group, a statistically significant reduction in the level of blood lactate formation was achieved at all assessed levels of the 8 × 100 progressive test after 8 weeks of inspiratory muscle training. Furthermore, a significant improvement in the strength of the respiratory muscles was observed.

IMT may also lead to a reduction in HR, which is the result of the resistive load on the inspiratory muscles caused by the applied stimulus. The IMT-mediated reduction in HR may be primarily due to the increased oxidative capacity of the inspiratory muscles, which attenuates the accumulation of metabolites in the inspiratory muscles, thereby reducing diaphragmatic afferent and efferent type III and IV nerve endings [45]. In our study, a significant reduction in HR was observed only after swimming 100 m at speeds of 88% and 93% of the maximum speed in the group performing IMT at the level of 50% of the MIP. At the same time, it is worth emphasising the fact that the obtained effects persisted for 2 months after completing the inspiratory muscle training, which may indicate the high effectiveness of this form of training.

The current state of knowledge indicates that this type of training may reduce sympathetic nervous system activity by suppressing the metaboreflex in respiratory muscles and improving functional capacity and physical performance, heart rate variability (HRV) and heart rate [14,46,47]. The available literature indicates that the effect of low- and moderate-intensity IMT may be a reduction in heart rate (HR) and diastolic blood pressure (DBP) [46]. In our study, a reduction in HR was achieved only when measured after swimming 100 m at speeds of 88% and 93% of the maximum speed in the study group. It is also worth emphasising the fact that the obtained training effect was maintained in this group after the end of IMT.

Inspiratory muscle training with a load of 50% MIP significantly increased the respiratory muscle strength of swimmers with disabilities. The limitations of this work are presented in the Appendix A.

## 5. Conclusions

1. Inspiratory muscle training at 50% MIP significantly reduced the fatigue response to exercise as assessed by the Borg scale.
2. Inspiratory muscle training a higher-intensity training (50% MIP) level significantly improved the training level of the swimmers tested, which was associated with an increase in swimming distance. The results obtained confirm the validity of inspiratory muscle training among athletes with disabilities. The introduction of higher-intensity inspiratory muscle training significantly reduced the level of blood lactate formation in the progressive test, which authenticates the validity of an additional supplementation of the preparation of athletes with this type of training.
3. Higher-intensity training (50% MIP) significantly reduced the heart rate in the 8 × 100 progressive test at 88% and 93% of the maximum speed, thus improving the aerobic endurance of the athletes.
4. The use of inspiratory muscle training at 50% MIP effectively improved the training level and physiological parameters of the swimmers’ effort. This indicates the need to include this type of training in the standard of competitive preparation of athletes with disabilities.

## Figures and Tables

**Table 1:** The characteristics of the study groups.

Variable	Group I n = 8	Group II n = 8	t/χ2	*p*
Age [years]	16.75 ± 2.66	17.88 ± 4.39	−0.62	0.5452
Body mass [kg]	61.75 ± 11.18	61.38 ± 13.71	0.06	0.9530
Body height [m]	1.77 ± 0.09	1.69 ± 0.09	1.62	0.1278
BMI [kg/m^2^]	19.68 ± 1.76	21.24 ± 3.39	−1.16	0.2666
Sports experience [years]	8 ± 3	7 ± 2	1.08	0.2036
Sports achievements	World Cup medallist, senior men’s MP medallist—n = 7 finalist of Tokyo IP, 5th place at Senior World Championships in 400 m freestyle—n = 1 participant of Tokio IP—n = 1	World Cup medallist, senior men’s MP medallist—n = 7 participant in Tokyo IP, World Cup medallist—n = 1 Participant of Senior Men’s European Championships in 202—n = 1 Participant of Tokio IP—n = 1	-	-
Swim class	S10—n = 5 S9—n = 3 SB10—n = 4 SB9—n = 2 SB8—n = 2 SM10—n = 5 SM9—n = 3	S10—n = 1 S9—n = 4 S8—n = 1 S7—n = 2 SB9—n = 3 SB8—n = 3 SB6—n = 2 SM10—n = 1 SM9—n = 4 SM8—n = 1 SM7—n = 2	1901	-
Type of dysfunction (disability)	− amelia—congenital lack of hands—n = 1 − hemiplegia—light level of disability—n = 1 − hemiparesis on one side of body—n = 1 − congenital hypoplasia of upper limbs, limitation of mobility in lower limbs—n = 1 − limitation of mobility in upper limbs—n = 2 − restriction of mobility in lower limbs—n = 2	− amelia—congenital absence of limb at level of forearm n = 3 − MDP—diplegia lower limbs—n = 2 − unilateral arm amputation—n = 1 − peripheral arthrogryposis, limitation of mobility in limbs, limb deformities—n = 1 − muscle weakness in lower limbs—n = 1	3279	-

**Table 2:** Inspiratory muscle training program for group I.

Training Week	1	2	3	4	5	6	7	8
Training load (cm H_2_O)	20% MIP	30% MIP	30% MIP	40% MIP	40% MIP	50% MIP	50% MIP	50% MIP
Training session [seriesxnumber of breaths]	2 × 30	2 × 30	2 × 30	2 × 30	2 × 30	2 × 30	2 × 30	2 × 30

**Table 3:** Inspiratory muscle training program for group II.

Training Week	1	2	3	4	5	6	7	8
Training load (cm H_2_O)	15% MIP							
Training session [seriesnumber of breaths]	1 × 60							

**Table 4:** The mean values and standard deviations of the maximal inspiratory and expiratory pressures in the study groups.

Variables	Test I		Test II		Test III	
	Group I	Group II	Group I	Group II	Group I	Group II
MIP [kPa]	10.38 ± 2.49	9.86 ± 3.17	14.21 ± 2.97	9.76 ± 2.93	12.77 ± 3.16	9.61 ± 2.85
MIP [%]	96.37 ± 21.31	96.35 ± 18.24	132.03 ± 25.24	94.95 ± 14.94	118.96 ± 25.92	93.95 ± 14.29
MEP [kPa]	10.87 ± 2.35	9.25 ± 3.84	13.06 ± 2.57	9.43 ± 4.38	12.58 ± 3.80	9.26 ± 4.21
MEP [%]	80.70 ± 13.92	72.52 ± 23.55	98.60 ± 23.43	73.76 ± 27.02	94.55 ± 24.70	72.66 ± 25.68

Abbreviations: MIP—maximal inspiratory pressure; MEP—maximal expiratory pressure.

**Table 5:** The variation in the mean values of the tested parameters of the maximum inspiratory and expiratory pressures. An analysis of variance for repeated measurements—probabilities for post hoc tests, LSD test.

Variables	Tests I–II in Group I	Tests I–II in Group II	Tests I–III in Group I	Tests I–III in Group II	Tests II–III in Group I	Tests II–III in Group II	Tests II–III in Group II	Test II in Group I and Test II in Group II	Test III in Group I and Test III in Group II
MIP [kPa]	0.0000	0.8559	0.0001	0.6413	0.0121	0.7757	0.7258	0.0076	0.0460
MIP [%]	0.0000	0.7832	0.0001	0.6381	0.0152	0.8447	0.9985	0.0018	0.0244
MEP [kPa]	0.0081	0.8105	0.0340	0.9846	0.5388	0.8255	0.3816	0.0598	0.0825
MEP [%]	0.0061	0.8380	0.0295	0.9808	0.5067	0.8568	0.4930	0.0466	0.0765

**Table 6:** The mean values and standard deviations of the assessed parameters in the studied groups.

Variables	Test I		Test II		Test III	
	Group I	Group II	Group I	Group II	Group I	Group II
T-30 [m]	1902.50 ± 187.45	1643.75 ± 461.33	1960.00 ± 207.83	1635.63 ± 399.12	1940.00 ± 204.28	1632.50 ± 401.17
Borg scale—RPE scale	12.88 ± 0.99	13.13 ± 1.13	11.88 ± 1.13	13.50 ± 1.2	12.00 ± 0.93	13.88 ± 1.13
La [mmol] d-77%	4.05 ± 2.17	3.54 ± 1.39	3.69 ± 1.99	3.74 ± 1.09	4.00 ± 2.11	3.65 ± 1.30
La [mmol] d-83%	4.99 ± 2.97	3.91 ± 1.77	4.81 ± 2.93	4.19 ± 1.69	4.91 ± 2.93	4.09 ± 1.60
La [mmol] d-88%	5.95 ± 2.99	4.55 ± 1.57	5.69 ± 2.82	4.69 ± 1.46	5.80 ± 2.94	4.64 ± 1.56
La [mmol] d-93%	9.96 ± 3.54	7.10 ± 1.85	9.83 ± 3.38	7.10 ± 1.81	9.89 ± 3.47	7.23 ± 1.90
La [mmol] d-100%	15.91 ± 2.51	15.59 ± 2.59	15.74 ± 2.40	15.70 ± 2.58	15.84 ± 2.44	15.71 ± 2.60
HR d-77%	122.50 ± 9.55	121.88 ± 13.83	121.25 ± 8.78	125.50 ± 8.88	123.00 ± 8.57	123.88 ± 12.30
HR d-83%	140.25 ± 6.71	134.38 ± 9.97	138.25 ± 7.03	137.63 ± 8.96	146.88 ± 21.64	135.63 ± 8.48
HR d-88%	150.00 ± 7.87	143.00 ± 8.32	148.38 ± 7.42	143.75 ± 8.10	148.75 ± 7.89	143.88 ± 8.43
HR d-93%	163.00 ± 9.96	158.25 ± 11.59	162.00 ± 9.47	159.25 ± 11.65	162.25 ± 9.60	159.25 ± 11.65
HR d-100%	180.88 ± 8.29	183.63 ± 8.93	179.88 ± 7.74	183.88 ± 7.70	180.13 ± 8.37	184.63 ± 9.24

T-30—30 min swimming test; Borg scale—RPE scale—Borg Rating of Perceived Exertion scale; La [mmol] d-77%—blood lactate formation concentration after swimming 100 m at 77% of maximum speed; La [mmol] d-83%—blood lactate formation concentration after swimming 100 m at 83% of maximum speed; La [mmol] d-88%—blood lactate formation concentration after swimming 100 m at 88% of maximum speed; La [mmol] d-93%—blood lactate formation concentration after swimming 100 m at 93% of maximum speed; La [mmol] d-100%—blood lactate formation concentration after swimming 100 m at maximum speed; HR d-77%—heart rate after swimming 100 m at 77% of maximum speed; HR d-83%—heart rate after swimming 100 m at 83% of maximum speed; HR d-88%—heart rate after swimming 100 m at 88% of maximum speed; HR d-93%—heart rate after swimming 100 m at 93% of maximum speed; HR d-100%—heart rate after swimming 100 m at 77% at maximum speed.

**Table 7:** Variations in the average values of the tested parameters. An analysis of variance for repeated measurements—probabilities for post hoc tests, LSD test.

Variables	Tests I–II in Group I	Tests I–II in Group II	Tests I–III in Group I	Tests I–III in Group II	Tests II–III in Group I	Tests II–III in Group II	Test I in Group I and Test I in Group II	Test II in Group I and Test II in Group II	Test III in Group I and Test III in Group II
T-30 (1) [m]	0.0020	0.6337	0.0344	0.5101	0.2455	0.8543	0.1388	0.0691	0.0831
Borg RPE scale	0.0054	0.2680	0.0135	0.0318	0.7092	0.2680	0.6493	0.0064	0.0021
La (1) [mmol] d-77%	0.0006	0.0409	0.5963	0.2380	0.0023	0.3564	0.5627	0.9547	0.6917
La (1) [mmol] d-83%	0.0259	0.0009	0.3221	0.0259	0.1897	0.1897	0.3852	0.6104	0.5027
La (1) [mmol] d-88%	0.0001	0.0252	0.0154	0.1434	0.0631	0.3969	0.2490	0.4048	0.3349
La (1) [mmol] d-93%	0.0089	1.0000	0.1361	0.0162	0.2114	0.0162	0.0585	0.0700	0.0760
La (1) [mmol] d-100%	0.0043	0.0552	0.1930	0.0345	0.0862	0.8257	0.8002	0.9767	0.9224
HR (1) d-77%	0.3030	0.0050	0.6779	0.1043	0.1529	0.1834	0.9069	0.4314	0.8700
HR (1) d-83%	0.6536	0.4671	0.1441	0.7789	0.0605	0.6536	0.3210	0.9152	0.0627
HR(1) d-88%	0.0000	0.0307	0.0007	0.0129	0.2649	0.7074	0.1022	0.2674	0.2435
HR(1) d-93%	0.0015	0.0015	0.0132	0.0015	0.3853	1.0000	0.3894	0.6151	0.5837
HR(1) d-100%	0.0608	0.6290	0.1539	0.0608	0.6290	0.1539	0.5229	0.3566	0.3016

## Data Availability

The data presented in this study are available on request from the corresponding author.

## Appendix A

Limitation: This study was conducted on a relatively small group including one national representation. Therefore, further studies should consider verifying long-term effects using a larger sample and a longer follow-up period. Future studies should consider increasing the group by including representation from other countries. In addition, it would be useful to supplement the assessment of the effectiveness of inspiratory muscle training with the verification of respiratory functions such as diaphragm excursion and/or thickening.
